# The living dead? Perception of persons in the unresponsive wakefulness syndrome in Germany compared to the USA

**DOI:** 10.1186/s40359-018-0217-4

**Published:** 2018-02-21

**Authors:** Inga Steppacher, Johanna Kissler

**Affiliations:** Department of Psychology, University of Bielefeld, Universitätsstr. 25, 33615 Bielefeld, Germany

**Keywords:** Mind perception, Morality, End of life decisions, Dualism, Disorders of consciousness

## Abstract

**Background:**

The extent to which people ascribe mind to others has been shown to predict the extent to which human rights are conferred. Therefore, in the context of disorders of consciousness (DOC), mind ascription can influence end of life decisions. A previous US-American study indicated that participants ascribed even less mind to patients with unresponsive-wakefulness-syndrome (UWS) than to the dead. Results were explained in terms of implicit dualism and religious beliefs, as highly religious people ascribed least mind to UWS. Here, we addresses mind ascription to UWS patients in Germany.

**Methods:**

We investigate the perception of UWS patients in a large German sample (*N* = 910) and compare the results to the previous US data, addressing possible cultural differences. We further assess effects of medical expertise, age, gender, socio-economic status and subjective knowledge about UWS in the German sample.

**Results:**

Unlike the US sample, German participants did not perceive UWS patients as “more dead than dead”, ascribing either equal (on 3 of 5 items) or more (on 2 items) mental abilities to UWS patients than to the dead. Likewise, an effect of implicit dualism was not replicated and German medically trained participants ascribed more capabilities to UWS patients than did a non-medical sample. Within the German sample, age, gender, religiosity and socio-economic status explained about 15% of the variability of mind ascription. Age and religiosity were individually significant predictors, younger and more religious people ascribing more mind. Gender had no effect.

**Conclusion:**

Results are consistent with cross-cultural differences in the perception of UWS between Germany and the USA, Germans ascribing more mind to UWS patients. The German sample ascribed as much or more but not less mind to a UWS patient than to a deceased, although within group variance was large, calling for further research. Mind ascription is vital, because, in times of declining resources for healthcare systems, and an increasing legalization of euthanasia, public opinion will influence UWS patients’ rights and whether ‘the right to die’ will be the only right conceded to them.

**Electronic supplementary material:**

The online version of this article (10.1186/s40359-018-0217-4) contains supplementary material, which is available to authorized users.

## Background

‘How shall we regard those in [permanent vegetative state]? They are periodically awake, and their bodies breathe and digest on their own. These traits bespeak life. Yet they are not conscious and never will be: subjectively, this is death’ [1] (p. 41).

Medical progress has provided the public with a clinical picture that seems to blur the line between life and death. Patients with the unresponsive wakefulness syndrome (UWS; [2]; former vegetative state, VS; [3, 4] are defined to have no self-awareness or conscious perception of their surroundings. Since the body is undoubtedly alive, whether or not the ‘person’ him- or herself still is seems hard to determine. Even relatives close to the patients can get confused about this issue. Holland et al., for example, report interviews with relatives of long-term UWS patients, showing that some relatives literally state that a patient is dead and alive in quick succession. For example, they quote ‘Brian’, a brother of a UWS patient with: ‘He’s already dead. The only reason he’s not dead is because his heart pumps […]’ [5] (p. 417).

Accordingly, there is an ongoing ethical debate whether or not UWS patients should be considered ‘persons’ [6] and although DiSilvestro et al. come to the conclusion that they should, others disagree [7], implying that there is uncertainty even among the scientific and (medical)-ethics community.

One possible explanation for this uncertainty of a UWS patient’s ontological state could lie in the criteria based upon which people tend to ascribe mind. As pointed out by Waytz [8], people ascribe mind to others (or even to objects) whenever they ascribe experience and agency. Here, agency is seen as the ability to exert willful behaviors beyond mere reflective or spontaneous movements which function on an automatic level. It has been suggested that, when it comes to UWS patients, many focus on the body of the patient and since these ‘bodies’ lack all signs of agency and as it remains unclear whether or not they are able to consciously experience anything, people may hesitate to ascribe a mind to UWS patients. This assumption has been backed up by a study of Gray and colleagues with the striking title: ‘More dead than dead’ [9], whose participants indeed ascribed less mind to a UWS patient than to a recently deceased person.

In this study, Gray and colleagues conducted a series of experiments to determine how much mind is ascribed to a UWS patient in comparison to either a healthy person or a deceased individual (experiment 1, *n* = 201). They also tested for religious beliefs as a variable potentially influencing mind ascription in their participants (experiment 2, *n* = 143). In a third experiment (*n* = 55), participants were asked to imagine to either die or fall into UWS themselves and were asked to rate how bad each of these outcomes would be for them and their families. Consistently, participants rated UWS patients to have less mind than the deceased. Religiosity and implicit dualism (the belief, that matter and spirit are separate from one another, irrespective of religiosity) were found to influence mind ascription in that religious persons and those holding implicit dualism, ascribed more mind to the dead. In general, participants rated UWS to be a state worse than death, both, for themselves and for their families.

If so, this could have complex and severe consequences for patients, because, as Waytz showed, with the ascription of mind, moral rights are also conferred [8]. Conversely, if a person is seen as relatively mindless, we also risk objectifying him or her, consequently denying human rights [6, 10, 11], because the patient is no longer seen as a ‘person’ at all [6]. Whereas from the scientific point of view, the ‘real’ mental status of a UWS patient cannot yet be conclusively determined [12–14], it seems safe to say, that any living brain should have the capacity to experience more than a dead brain. In this regard, many recent studies have shown that UWS patients can exhibit a considerable range of cerebral responses to external stimuli [15–19]. Therefore, although we are not aware of any replication, Gray et al.’s study suggests a widely held misperception that could be to the disadvantage of the UWS patients, particularly, given that misdiagnosis rates for these patients are very high [20, 21] and, although prognosis is generally rather poor [22, 23], unexpected recovery can occur [24–26].

Historically, it is known that in times of limited resources, terminal and severely ill patients’ right to live has been questioned [27, 28]. Strikingly, whenever there is a discussion concerning passive or even active euthanasia, UWS patients are recommended first [27, 29]. The most common argument used to justify euthanasia in general is salvation from unnecessary suffering [30], although this should exclude UWS patients from eligibility for euthanasia, since, if diagnosed correctly, the syndrome precludes suffering. Still, it seems that ‘the right to die’ is often seen as the only right left for these patients [28, 29]. In Europe, there have even been political efforts towards common regulations for passive euthanasia for UWS patients which failed due to ‘different traditions and cultures concerning the matter’ [31]. Thus, even in the Western world, different cultures may hold different beliefs about UWS patients. Given the very concerning results of the Gray et al. study with US participants and the constantly declining financial support and resources in the health systems of most Western countries, it is vital to investigate the public beliefs about UWS patients as well as the factors influencing these perceptions.

Here, we aim to replicate and extend the series of studies reported by Gray et al., with a large German sample. So far, no published replication of Gray et al. exists, precluding strong claims about the generality of the findings. However, although USA and Germany, as Western cultures, are similar in many ways, there are also substantial differences that might influence the perception of UWS itself as well as the perceived tragedy of the resulting situation. According to Hofstede [32, 33], there are mainly six dimensions that can be used to characterize different cultures (see Additional file 1: Table S1). In particular, the scales of ‘individualism - collectivism’ and ‘avoidance of uncertainty’, reveal substantial differences between Germany and the US. The USA scores highest among 76 countries on individualism and the society is quite tolerant against uncertainties (rank 64 of 76 countries). US Americans value the self determined ‘I’ and the right and ability to live a self determined life. Autonomy and self-actualization are ultimate goals and freedom an individualist’s ideal [33]. Americans value leisure time and the fulfillment of desires, ‘now’ over ‘then’, and are, in general, not too concerned about the future [32]. Therefore, UWS could be considered the exact opposite of the life values of US Americans.

For Germans, living in a more moderately individualist culture and being very avoidant of uncertainty, the situation could be different: UWS, although undoubtedly tragic, may interfere a little less with cultural values (since for example personal freedom and autonomy of the self are not valued as highly as in the USA). Furthermore, because they have little tolerance for uncertainties, Germans tend to think through most possibilities of ‘what could happen’ in life and have insurances for all eventualities [34]. German obligatory health insurance indeed covers the unlikely event of UWS. There is also a special “care allowance” (‘Pflegegeld’) that is paid to care-giving family members as well as access to highly professional care institutions or outpatient care services to disburden families (‘Bundesministerium für Gesundheit’: http://www.pflegestaerkungsgesetz.de/ 11.04.2017). Ironically, for Germans, ‘having things thought through’ and feeling prepared could, in the unlikely event of having to care for a UWS patient or becoming one oneself, help to reduce the perceived tragedy of the UWS situation. Therefore, UWS could be regarded as more aversive in the USA than in Germany.

According to Hofstede [32, 33], short-term oriented cultures like the USA are also characterized by the belief that matter and spirit/mind are separated. Thus, members of such cultures should hold both explicit religious beliefs and implicit dualism. Long-term oriented cultures like Germany, on the other hand, in general, believe that matter and mind are integrated (which still allows for explicit religiosity but reduces implicit dualism). Indeed, Gray et al. [9] clearly demonstrated a separation in the perception of mind and matter (ascribing mental abilities to the deceased and none to the UWS patient) for their US sample. According to the above reasoning, Germans may generally ascribe more mind to UWS patients, since a living body is less likely seen without mind.

Besides general cultural factors, experience with UWS might also affect mind ascriptions.

Therefore, we asked all participants whether or not they personally know a UWS patient to assess the influence of familiarity with the syndrome since our own clinical experience as well as some studies [35] have shown, that some caregivers hold hopes for their patients that are not always shared by the medical staff.

Therefore, we explicitly included medical staff into the survey who should have expert knowledge in the area and whom we expected to have had, on average, exposure to both, UWS patients and deceased. The perception of UWS patients by medical doctors is of special importance since they are often involved in end of life decisions. In fact, it has been shown that up to 70% of all deaths on the examined critical care units occur due to discontinued life support which was recommended by doctors due to an ‘unfavorable prognosis’ (which usually includes a UWS prognosis) [36, 37]. Whether or not they ascribe mind to the patients and whether or not they perceive UWS ‘as a faith worse than death’ [38] is therefore likely to influence the advice and guidance they offer the families of patients [27].

Finally, since little is known about the factors influencing UWS perception even within a society, we assessed the potential influence of general demographic and socio-economic variables on mind ascription to UWS patients as an exploratory analysis.

In sum, we expect our German sample to ascribe more mind to the UWS patients and rate the condition of being in UWS as less tragic than the US sample did. We further expect the religious Germans to ascribe more mind to the deceased than the irreligious participants, but we expect no effect of implicit dualism within the German sample. Lastly, we explore the effects of personal knowledge and medical expertise as well as demographic variables on mind ascription to UWS patients.

## Methods

We included all three experiments of Gray et al. [9] into a large on-line survey. Therefore, we translated the stories of David, who, after a car accident, was either alive (story 1), in UWS (story 2) or dead (story 3 and 4; the latter with a focus on the dead body in the morgue, referred to as the ‘corpse-condition’). The names of American cities were replaced with German ones, otherwise, no changes were made (for both, English and translated story-vignettes please see Additional file 2: Text S1 and Additional file 3: Text S2).

### Online-setting

Unlike Gray and colleagues, who ran their experiments 1 and 2 as paper and pencil versions and only experiment 3 on-line, here, all stories and questions were presented as an online survey. One of the four David-scenarios (life condition, UWS condition, death condition and corpse condition) were randomly assigned to each participant. After reading the short story, participants were asked to rate, on a response-scale from 1 (strongly disagree) to 7 (strongly agree), the mental abilities of David according to five statements (‘David can influence the outcome of situations’, ‘David knows right from wrong’, ‘he remembers the events of his life’, ‘has emotions and feelings’, ‘is aware of his environment’ and ‘has a personality’).

After completing this part, all participants were asked to imagine that they themselves would be involved in a car accident and would either die or become a long lasting UWS patient. They were then asked to rate on a scale from 1 (not bad at all) to 7 (extremely bad) how bad the respective outcome would be for a) themselves and b) for their families. To ensure that envisaged financial consequences of long time care or burial did not influence the rating, participants were told that all ensuing costs were covered by insurance. After that, as a manipulation check, participants were asked to state what happened to David in the story they read. The correct responses for the life-condition (‘alive’), for the UWS-condition (‘alive, with severe brain damage’), and for both dead-conditions (‘deceased’) had to be indicated. Participants who failed to answer correctly were later excluded from the analysis.

The manipulation check was followed by three questions about religiosity and how strongly they believe in life after death. Participants were also asked which religion they belong to. Then, participants answered some demographic questions regarding age, gender, and workplace. Finally, we asked three additional questions: “How much do you think you know about UWS?”, “I have a UWS patient within my circle of acquaintances?” And: “I have/had contact to a patient in UWS?” For question one answers ranged from: 1 (nothing at all) to 7 (very much); Question two and three were yes or no questions. To complete the whole survey, participants needed about 5 to 10 min.

### Participants

German participants were recruited from the University of Bielefeld, personal connections, via Link-posting on Facebook and Flyers in six different clinics within the area of Bielefeld, Paderborn and Bad Salzuflen, state of North Rhine-Westphalia. To test for cultural differences it is important to recruit representative samples of participants. Unfortunately, little information is given about Gray’s US sample. It is stated that the sample was recruited randomly from college and metro areas in New England, Amtrak stations and New York City parks for the paper and pencil tests (experiment 1, *n* = 201; experiment 2, *n* = 143) and from the on-line platform MTurk to take part in the online survey (experiment 3; *n* = 55).

In our study a total of 991 participants finished the questionnaire. Nine participants had to be excluded for being underaged, 72 participants had to be excluded because they failed the manipulation check and therefore seemed to be unable to remember what had happened to David in their story. This leaves 910 datasets for further analysis. Demographics can been seen in Table 1.

**Table 1:** Participants demographics

Mean age (Range)	45 (18–86 years)
Female (%) / Male (%)	580 (64%) / 329 (36%)
Work area: Students / Medical background / Other work area	319 / 177 / 412
Socio-economical status Students / Vocational training / Employed university graduates / Graduate professionals / Retirees / Others	344 / 257 / 106 / 132 / 45 / 26
Religiosity: Christians / Atheists / Buddhist / Muslim / Hindus / other	596 / 265 / 25 / 8 / 2 / 14

‘Students’ include pupils, trainees and students; vocational training include for example physiotherapists, nurses, cooks, hairdressers, kindergarten teachers; employed university graduates include for example psychologists employed in a clinic; professionals include for example medical doctors and professors; retirees include all retirees that did not give specifics about previous employment. Classification following [58] recommendations for SES assessment in Germany

## Results

Some of the data violated the sphericity requirement (Levene-test). However, non-parametric testing which was also performed only leads to numerical but not qualitative changes. Therefore, in the following, we report parametric tests, to facilitate direct comparison with the original study.

Table 2 shows results of the random assignment of the four vignette conditions to the participants.

**Table 2:** Random vignette assignment

Condition	Number of Participants
Death	206 (23%)
Corpse	224 (25%)
UWS	223 (24%)
Life	257 (28%)
Total	910 (100%)

Expected cell frequency: 227.5; Chi-square test shows no significant deviation χ2 (3, *N* = 910) = 6.00, *p* = 0.11

### Mind perception

**Fig. 1:**
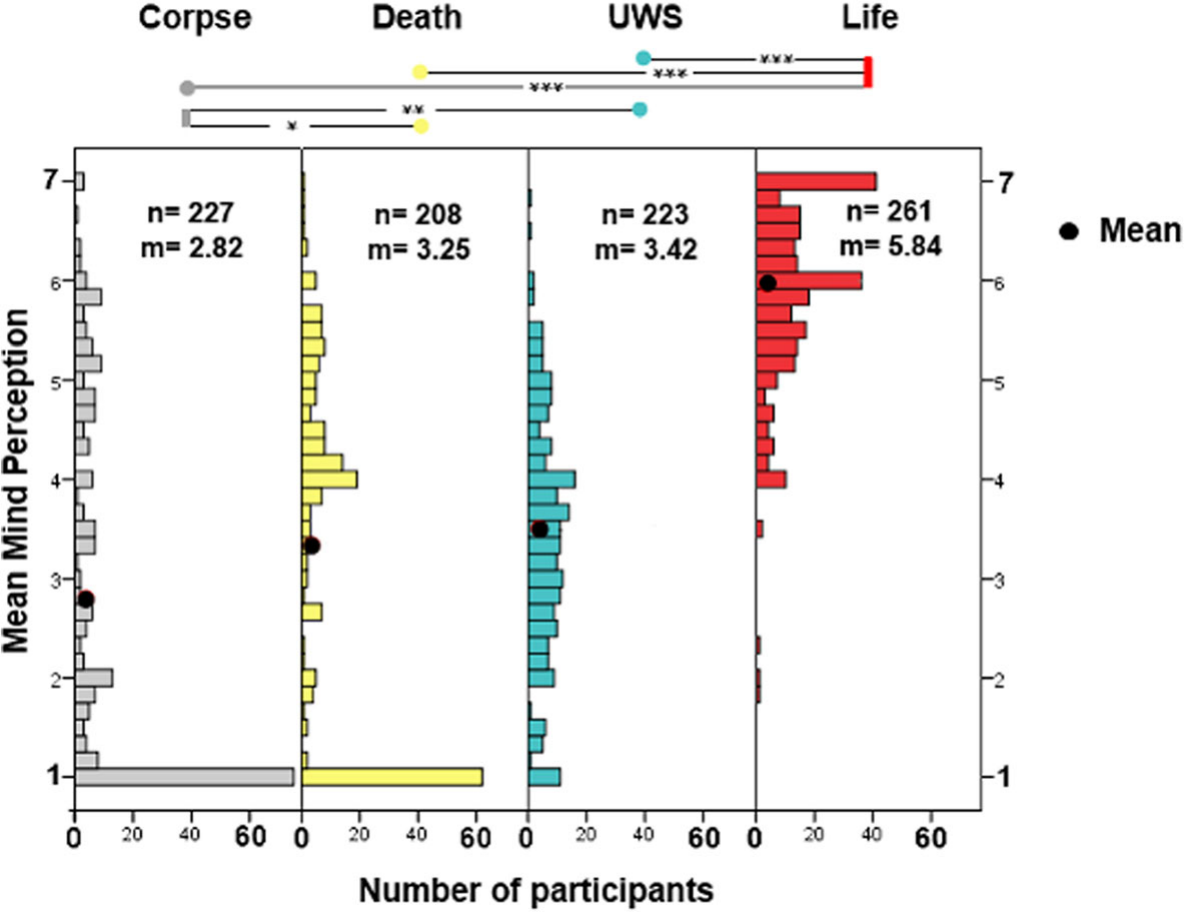
Mean mind perception of the participants in the four conditions where David died and the focus of the vignette lay on the dead body (Corpse), where David died (Death), where David survived but entered UWS (UWS) and where David survived with no further consequences (Life). Black points are mean values, bars represent the number of participants with respective mean mind perception score

As in Gray et al., a ‘mind-perception-index’ was formed by averaging the six mind perception questions. These indices of all four vignettes were submitted to an analysis of variance (ANOVA) which showed a significant effect for condition F(3, 909) = 204.34; *p* < .001. As Gray et al., we used Fisher’s least significant difference (LSD) post hoc test, which showed that the life condition differs significantly from every other condition (*p* < .001). There are also significant differences between the corpse condition and the death condition (*p* = .005) as well as between corpse and UWS (*p* < .001). UWS and Death conditions do not differ from each other. However, although mean values do not vary much, as evident from the histogram plot, there are substantially fewer participants completely denying mental life to the UWS patients than for both death groups (see Fig. 1).

**Fig. 2:**
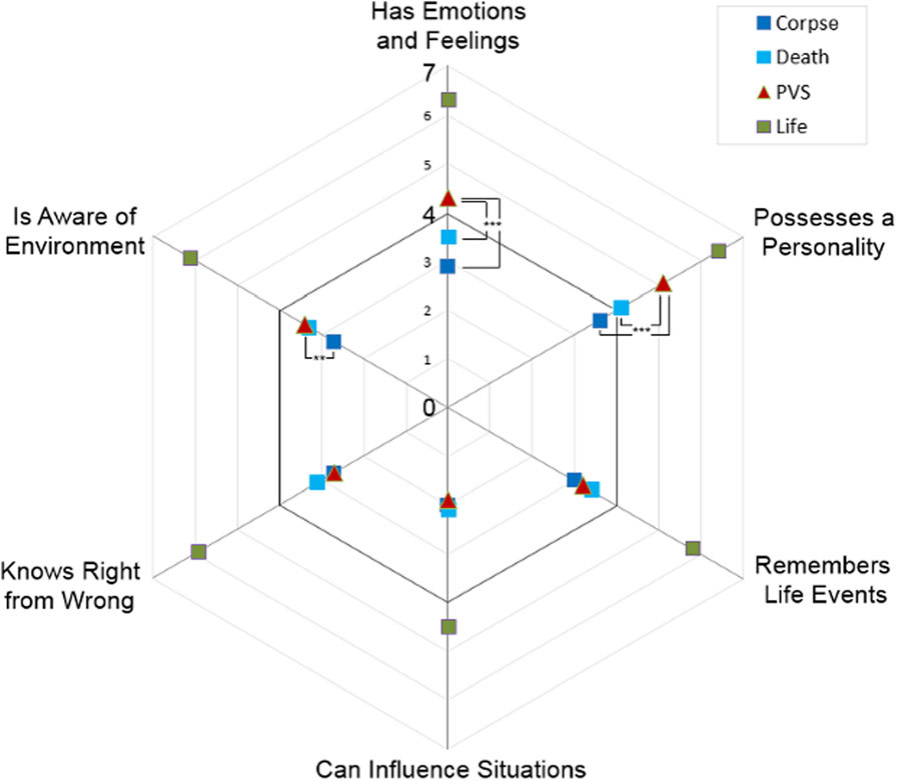
Perception of mind on individual items for all four vignettes. Answers above 4 refer to agreement with the item, 4 is neutral, under 4 indicates disagreement

Like Gray et al., we also analyzed the individual items. It was confirmed that participants in the life condition ascribe the most mental capabilities on every item (*p* < 0.001). But unlike in the study of Gray and colleagues, UWS-David compared to the David in both death conditions was ascribed significantly more personality and is perceived as possessing more emotions and feelings (see Fig. 2).

### Mean mind ascription: present data versus Gray et al

**Fig. 3:**
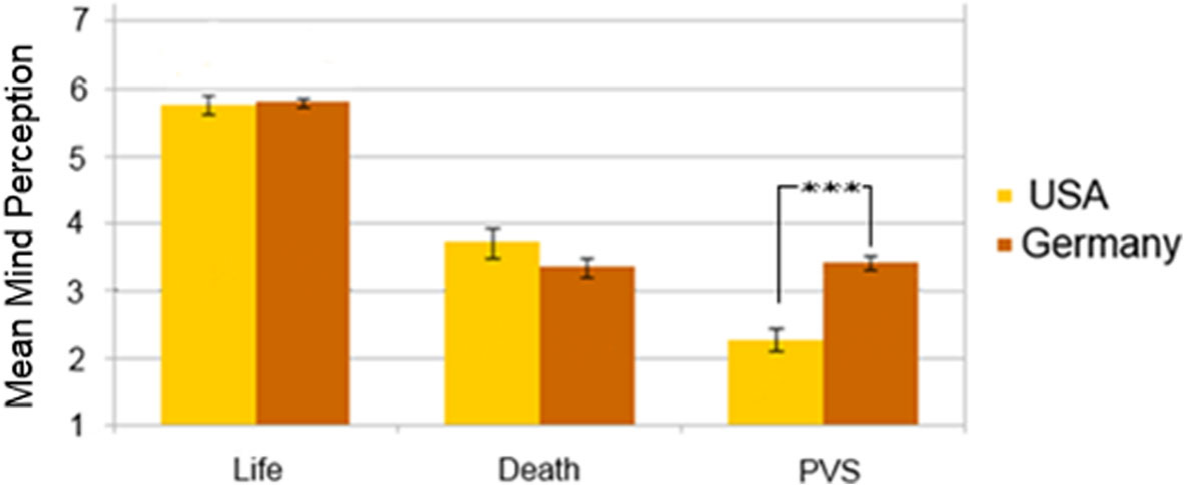
Differences of mind ascription between the conditions life, death and UWS in USA and Germany. Error bars are +/− one standard deviation

To compare Germany with the USA, the USA means and standard-deviations were extracted from Gray et al. and transformed to the same scale (from 1 to 7). There is no significant difference between either the life conditions assessment of the American participants (*n* = 67, M = 5.77, SD = 1.76) and the German ones (*n* = 257, M = 5.83, SD = 0.95) or for the death condition in the USA (*n* = 67, M = 3.71, SD = 1.76) versus Germany (*n* = 206, M = 3.23, SD = 1.80). There is, however, a highly significant difference between the ascription of mind for the UWS condition in the USA (*n* = 67, M = 2.27, SD = 1.36) and Germany (*n* = 223, M = 3.42, SD = 1.25), t(288) = 6.47, *p* < 0.001 (see also Fig. 3), with US participants ascribing less mind to the UWS patient than Germans do. There is also an overall difference between the death and UWS condition occurring only within the US sample. Due to the fact that mean and standard deviation for the corpse condition were not reported in the US study, a cross-cultural comparison between these two conditions could not be calculated.

### Religiosity

**Fig. 4:**
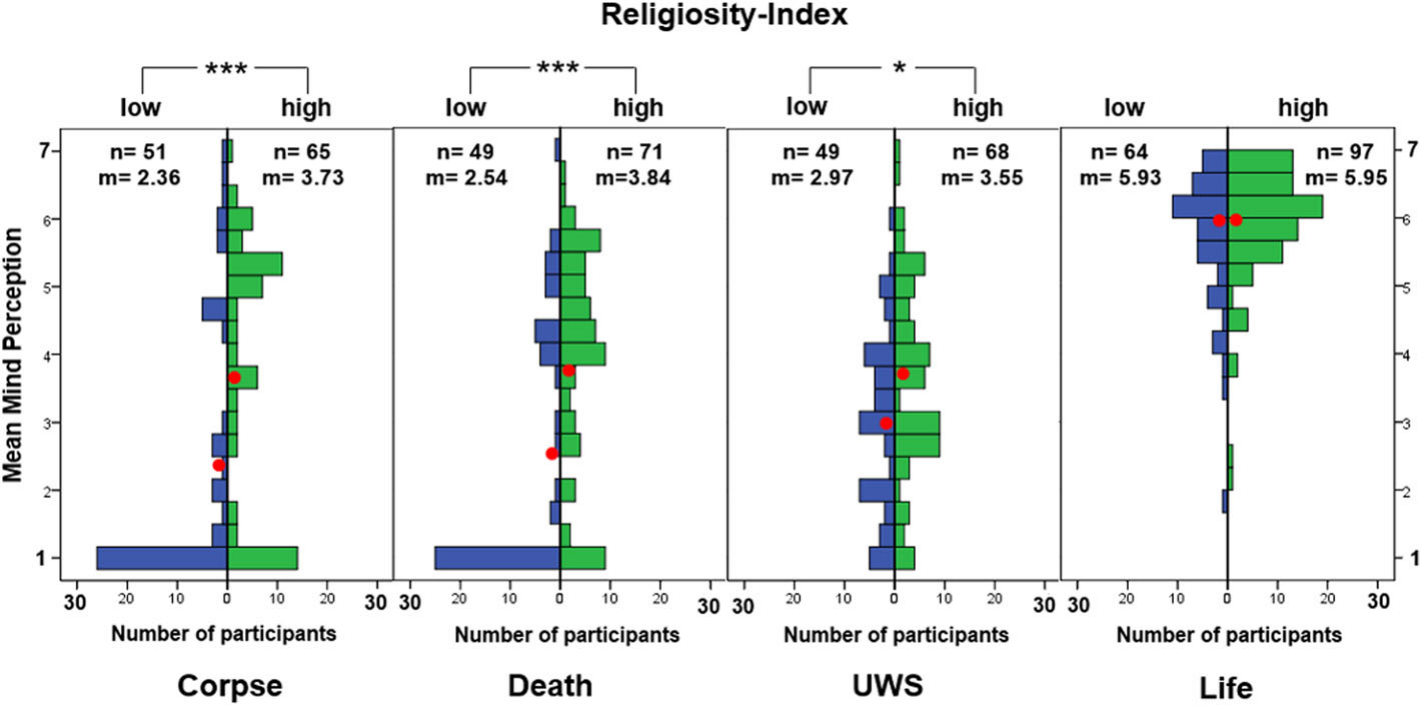
Histogram for participants with a high and low religiosity-index. Displayed are mean mind perception scores (red point) for the four conditions. Bars represent the number of participants with respective mean mind perception scores

Following Gray et al., we averaged the religiosity items to form a religiosity index. We then split all participants into thirds according to their scores on this index and compared those who scored at the top and the bottom third of the scale (tertiary split, [39]). This results in *n* = 211 (23%) participants with a low religiosity score and *n* = 298 (33%) with a high score. Four hundred one participants with medium religiosity scores were excluded from this analysis. We examined differences on the mind perception index in a 4 (dead, corpse, UWS, life) × 2 (religiosity high/low) ANOVA. There were significant main effects for condition F(3, 501) = 123.53, *p* < 0.001, ɳ^2^ = 0.43; for religiosity F(1, 501) = 35.90, *p* < 0.001, ɳ^2^ = 0.07 (with religious participants ascribing more mind in general) as well as a small but still highly significant interaction between the two factors F(3, 501) = 5.67, *p* = 0.001, ɳ^2^ = 0.03, essentially resulting from the fact that a group difference occurred in all conditions except the life condition (see Fig. 4).

Within both groups, mind perception scores are higher in the Life than in all other conditions *p* < 0.001. Corpse, Death and UWS conditions do not differ within either group.

Between groups, the corpse conditions differ significantly (*p* < 0.001), as does the death (*p* < 0.001) and the UWS condition (*p* = 0.026). In each case, religious participants gave, on average, higher scores than non-religious ones. There is no between-group difference for the life condition.

### Severity of outcome: present data versus Gray et al

To compare the severity of outcome as perceived by US and German participants we used the mean values of the death scale as a reference. We then calculated the difference for both groups of how much worse or how much better than death, UWS was perceived. This revealed the fact, that, on average, US participants perceived UWS as 1.52 points (on a 7 point scale) worse than death. Germans feel, on average, that UWS is 0.39 points worse than death. The independent samples t-test showed that both groups vary highly significant in the perceived severity of the situation t(223) = 3.18; *p* = 0.002. We used the same difference measure for when participants considered how bad this outcome would be for their families. Here, US participants rated to become a UWS patient to be 1.04 points worse for their families than an early death. Germans rated becoming a UWS patient as 0.01 points better than an early death. Again, the difference between both ratings is highly significant t (223) = 5.22; *p* < 0.001.

Additionally, in the German sample, we found a significant correlation (r(223) = .25, *p* < 0.001) between the mind perception score and the evaluation of the badness of the situation. It has, however, the opposite direction than in Gray et al. where less mind was ascribed, the worse the condition is viewed. Here we found, that the more mind is ascribed, the worse the condition is viewed.

### Subjective knowledge and exposure to UWS patients

Correlations within the UWS vignette revealed no correlation between either subjective knowledge about UWS and mind ascription (r(218) = −.06, *p* = 0.422) or real world experience with UWS patients and mind ascription (point-bi-serial correlation for ‘I have a UWS patient within my circle of acquaintances’ (Yes/No): (r(218) = − 10, *p* = 0.138) and for I have/had contact to a UWS patient: (r(218) = −.06, *p* = 0.394)).

### Medical background

**Fig. 5:**
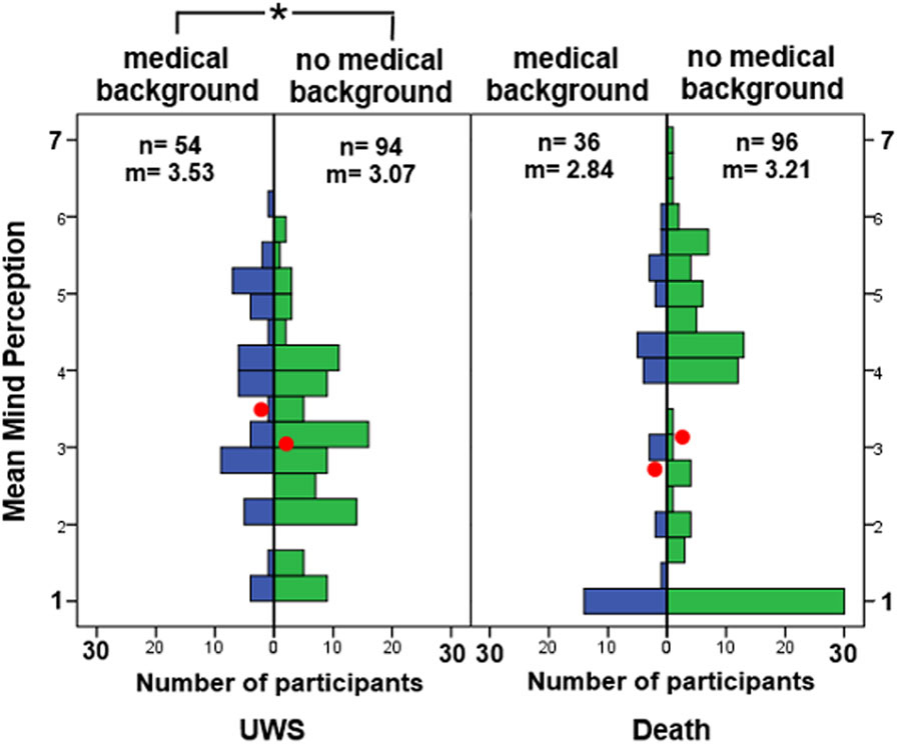
Differences in mind ascription from employed participants with and without medical background for the UWS and death vignette. Displayed are mean mind perception scores (red point) for the two conditions. Bars represent the number of participants with respective mean mind perception score

Since we were especially interested in the perception by medical staff of UWS patients and the dead, we compared the mind ascription of participants with a medical background and other participants specifically within the UWS and Death condition. For better comparability and in an attempt to exclude other factors like general life experience, work situation and family status, for this comparison we excluded all students and trainees from both groups. Independent samples t-Test within the UWS vignette reveals a significant difference between participants with a medical background (*n* = 54, M = 3.53, SD = 1.30) and other employed participants without a medical background (*n* = 94, M = 3.07, SD = 1.17), (t(158) = − 2.65, *p* = 0.03), participants with medical background ascribing more mental capabilities to UWS patients. For the death vignette there is no such difference (medical background: *n* = 36, M = 2.84, SD = 1.78; no medical background: *n* = 96, M = 3.21, SD = 1.83) t(130) = − 1.04, *p* = 0.30 (see also Fig. 5).

To further analyze the effect of medical expertise for perception of UWS, an additional sample of medical professionals was collected. Since the 54 medical professionals originally assigned to the UWS condition do not allow for further separations of professions, we additionally recruited further medical professionals in a second recruitment at the Kliniken Schmieder, Allensbach, Germany. Because these participants where added posthoc, they were not included into any analysis other than this one. This resulted in *n* = 71 medical professionals for this analysis in the PVS condition. Seven participants had to be excluded due to the fact that the profession was too rare (for example midwife or pharmacist) for further analysis. One person had to be excluded because he stated to work in the medical field but failed to identify his profession. Further descriptive characteristics are detailed in Table 3. One-way ANOVA revealed no significant differences in mean mind ascription between the medical groups; F(3, 57) = .959; *p* = .418. As can be seen in Table 3, medical doctors do, on average, ascribe as much mind as do participants with no medical background and the effect that persons with medical background ascribe more mind seems to be mainly driven by the other medical professions. However, if tested with t-test for medical doctors vs. all other medical professions, there is a trend, but no significant difference between professions; t(61) = 1.76; *p* = .084.

**Table 3:** descriptive statistics for medical professionals in general mind ascription

	Mean	Std. Deviation	N
Medical doctors	2.89	1.52	15
Nurses / paramedics	3.75	1.00	21
Doctor’s assistants	3.40	1.32	13
Therapists /psychologists	3.47	1.16	14
Total	3.41	1.24	63

We were further interested in whether doctors perceive UWS as a state worse than death. We therefore compared 15 doctors from the UWS condition with 22 doctors within the death and corpse condition. Independent t-test showed that doctors perceive UWS as equally tragic (M = 6.00) as an early death (M = 5.05); t(35) = 1.16; *p* = .256 for themselves and for their families (UWS M = 6.2; early death M = 6.77; t(35) = 1.42; *p* = .164).

### Regression model

Lastly, we performed a stepwise backward regression analysis into which we entered the factors: age (as a continuous variable), gender (coded with 0/1), subjective knowledge (ranging from 0 to 7), religiosity index (ranging from 0 to 7) and socio-economical status (dummy coded). Backward exclusion resulted in a model that included the factors age, religiosity and socio economic status. The factors gender and subjective knowledge were excluded, since their removal did not significantly reduce the variance explained by the regression model. The final model was highly significant (F (7/210) = 5.39; *p* < 0.001). Together, the factors are able to explain 15.2% of the variance in mind ascription for UWS David (see Table 4). Age and religiosity were individually significant, younger people and more religious people ascribing more mind, whereas none of the socio economic status categories had an individual effect.

**Table 4:** Stepwise backward regression model

	*B*	*SE B*	β	t
Constant	4.66	.78		5.99***
Age	−0.02	.01	−.39	−3.84***
Religiosity index	0.10	.05	.14	2.14*
Socio-economic status				
Students	−0.77	.72	−.30	−1.07
Vocational training	−0.39	.69	−.15	−0.57
Employed university graduates	−0.77	.70	−.22	−1.10
Graduate Professionals	−0.54	.70	−.15	−0.77
Retirees	−0.25	.80	−.04	−0.31

R^2^ = .15; *p* < 0.05 *, *p* < 0.01 **, *p* < 0.001***

## Discussion

We assessed to what extent German participants ascribe mental functions to a UWS patient compared to a deceased or a healthy person, what factors influence this ascription, and how the results compare to a previous US study. In particular, we analyzed the possible role of cultural differences and religiosity in comparison to the US study, as well as the influence of age, gender, socio-demographics, knowledge about and familiarity with UWS patients and medical expertise in the German sample.

General results showed that German participants ascribe as much or more, but not less mental abilities to UWS patients than to the dead. On the single item basis they ascribe significantly more ‘emotions and feelings’, as well as ‘personality’ to a UWS patient. Additionally, it is worth noticing, that in the present study participants tend to disagree with all mind-items for the dead person (dead and corpse condition; means under 4 which indicates that most participants disagree with the item), while they endorse that UWS patients have ‘emotions and feelings’ as well as that they possess a personality (means over 4, indicating agreement). Furthermore, and in line with cultural difference, the mean mind ascriptions differ significantly between Germany and the USA, with the German participants viewing UWS as a state of life rather than ‘something less than dead’ [9] (p.278). So, from our results, it seems that Germans indeed hesitate to see a living body as mindless, since the living, but injured brain was less likely seen without mind. However, as obvious from Fig. 1, mind ascription to a UWS patients varies greatly between participants.

We also found, that the Germans find the situation significantly less tragic for themselves and for their families than the US sample does. This was predicted by differences between German and American cultures, mainly on the ‘individualism’, scale by Hofstede [32, 40], which implies that for the US, as the most individualistic culture, the loss of autonomy and self-determination should result in a most aversive situation.

To explain the generally low mind ascription to a UWS patient and the astonishingly high mind ascription to a dead person within the US sample, Gray argued that the ‘apparent reasons for such perceptions are afterlife beliefs and the tendency to focus on the bodies of UWS patients (Experiment 2)’ [9], (p. 278). Regarding the other conditions, we found, that religious participants tend to ascribe more mind in all but the life condition. This is in line with Demertzi [41] who found in her study that religion was the best predictor for the participants’ answers, ‘Yes, the UWS patient can feel pain’ which means that religious participants were more likely to ascribe a specific subjective experience to the patient than non-religious participants were. However, as predicted, in our study implicit dualism seems to play no important role for ascribing mind to the dead since mind ascription did not drop significantly in the corpse condition for low religious Germans. Furthermore, if implicit dualism were the explanation, Germans would ascribe fewer mental abilities to the dead than Americans do, which was not the case. Germans ascribed selectively more abilities to the UWS patient than Americans did.

Therefore, implicit dualism seems indeed not as widespread a phenomenon in Germany as it is in USA. However, at least for some German participants the line between life and death for these patients may likewise be somewhat blurred. Concerning the manipulation check, we excluded 72 participants who had given the wrong answerer to the question of what happened to David in the vignette story. In the death vignette one was excluded because he stated that David was alive, 9 thought he had survived with severe brain damage (which would be the correct answer for the PVS vignette). In the life vignette 15 were excluded because they said David survived with severe brain damage. In the PVS vignette however, 43 participants (42,5% of all participants failing the manipulation check) answered that David had died. Fischer’s exact test confirms that this is significantly more than in the other vignettes (*p* < 0.000). Given the description of the PVS vignette with the very severe brain damage and no hope of recovery, stating that David was dead might not have been a real mistake. Maybe some of the participants actually thought that, while the body was still alive, the person David had died. This is of course a speculation, but if so, the practice of excluding these participants from the study might have actually excluded mostly participants that engage in active dualism. Further studies could address this issue by asking the participants to explain their choice of answer.

Differences in the amount of dualism in different societies are in fact documented within previous studies: For example, dualism is widespread in the USA [42, 43], whereas Demertzi and colleagues [44] comparing dualism beliefs between an Edinburgh-sample (Scotland) and a Liège-sample (Belgium) found substantial differences, with dualism being significantly more common in Edinburgh. To the best of our knowledge, no study has directly compared dualism beliefs for USA and Germany so far, but the more general role of religion has been subject to various studies. Verweij [45], for example, points out, that there is an ongoing secularization within all Western countries, except the United States - the only Western culture relatively untouched by secularization.

Gray also argued that ‘there may also be other variables operating in perception of UWS patients, such as liking and familiarity’ [9], (p.279). Very recent studies seems to confirm that since Moretta et al. demonstrated, that patients caregivers tend to ascribe more interaction abilities to their relatives than physicians do [46]. However, in our study neither familiarity with nor the subjective knowledge about UWS patients influences the mind perception of the participants. There is, however, an effect for a medical background. Gray speculated that it might be possible that ‘even doctors may see UWS patients as having less mind than the dead’ [9] (p.279), a conclusion drawn from the fact that many health care professionals, at least in Belgium, also advocate dualism [41]. In our sample, participants with a medical background were explicitly included. This covers physicians as well as nurses, medical technical assistants, physiotherapists, psychologists and paramedics. Mind ascription differed specifically in the UWS condition, with participants with medical background ascribing, contrary to Gray’s hypothesis, significantly more mental capacity to UWS patients than to the dead. Similarly, Demertzi asked European paramedical caregivers and medical doctors whether or not they think that a UWS patient can experience pain: Here, about 60% of the participants answered with ‘Yes’, therefore ascribing this mental ability to the patient [41]. Kuehlmeyer and colleagues investigated the same question in German and Canadian physicians and here as many as 70% ascribed the ability to feel pain to UWS patients. Another 51% believed that patients are able to feel touch and 21% of the physicians even were convinced that UWS patients can experience dreams [47].

It has been argued that the contact with the patients increases the likelihood of ascribing mental capacities [48]. This could explain the present tendency for nurses and therapeutic professions, who usually spend more time with the patients than doctors, to ascribe the most mind, whereas doctors, on average, ascribe as much mind as our non-medical participants. However, this explanation would suggest that participants who personally know and in particular care for a UWS patient should also ascribe more mind, which was not observed for the non-medically trained participants. This contradicts the explanation that it is the mere exposure and time spent that leads to a higher mind ascription. A possible explanation would be, that participants with medical background often know more than one patient, resulting in more experience with the variability of brain functions, recovery, conscious experience and survival. They might also know more about scientific studies that indicate very high rates of false diagnoses [21, 49] as well as brain activity in UWS and minimally consciousness state [50–52], which have revealed conscious perception in patients that seem completely unresponsive at bedside examination. In fact, for example Yu et al. [19] found that the majority of UWS patients respond to other people’s cries of suffering, thus revealing some kind of emotional responses. Such findings might lead medical staff to give patients the benefit of the doubt. Overall, present response patterns are in line with the finding, that medical staff usually demonstrates much more negative attitudes toward active euthanasia than the lay population [30, 53, 54].

Another difference between the German and the US study is the correlation between perceived tragedy of the situation and mind ascription. In the USA people find the state of UWS the more adverse the less mind they ascribe to the patients, whereas in Germany, the opposite is true. This could result from the general value system in which the two correlated variables are embedded [55]. Coming from the most individualistic culture, US participants might prefer the possibility of conscious experience (which includes suffering) over experiencing nothing. In Germany, as a less individualistic culture, the correlation is also significant but in the opposite direction, suggesting that Germans perceive UWS as more tragic when ascribing more mind - maybe taking into account that with more mental abilities the possibility of conscious suffering also increases. If this is indeed a cultural phenomenon, than it would be interesting to see whether this judgment becomes even more pronounced within collectivist and perhaps particularly Buddhist societies. However, it is also possible, that the specific history of Germany (where during the Third Reich disabled persons were viewed as ‘unworthy of life’ and mass “euthanasia” was performed) could make Germans more reluctant to value any life as worse than death. If so, other otherwise similar societies (e.g. Swiss or perhaps French) with no history of nationalism should rate the tragedy of the situation more like the US participants.

In general, and in detail pointed out by Gomes and Parrott [56], there are some complications with the wording of the UWS vignette itself, such as the detailed description of a completely destroyed brain and the quotation marks on the used term: technically ‘alive’ in the UWS vignette. We adopted both in our translations. The very description of David’s state could make it hard for participants to ascribe any mental abilities to David and may test the participants’ intuition about brain functions more than their intuitive beliefs about UWS [56].

Another concern with wording results from the translation into German: We cannot be sure that the German term ‘Wachkoma’ (waking-coma), which is the commonly used term for UWS among the German population at large, triggers the same context for Germans as does ‘vegetative state’ in US Americans. ‘Wachkoma’ might have a more transient connotation for Germans than ‘vegetative state’ has for Americans. ‘Wachkoma’ also contains the word ‘(a)wake’ which implies an improvement to the coma-condition, focusing on the ability of the patients to open their eyes. The ‘vegetative’ in vegetative state, on the other hand, focuses on the inabilities of the patient and his or her “vegetative condition” which might evoke, and has been suggested to bring up, associations of vegetable-likeness [2]. This, in turn might have triggered different mind - sets in participants which might have led to different evaluations of mind between German and American participants. However, the story itself does make it very clear, that David’s ‘Wachkoma’ was very futile in terms of outcome. Thus, further research should aim to avoid these methodological issues by assessing the contexts that are activated due to different term-translations which could also be found with other languages.

Moreover, due to different recruiting methods, we might have divergent selection biases in recruiting the two samples. Neither Gray’s nor our sampling rely solely on students but recruited participants also randomly at either public places (Gray) or via link circulation in social media (current study). It is conceivable that the paper-and-pencil questionnaires that Gray et al. used in two of their studies, differ as such from on-line questionnaires in a hitherto unknown way.

Regarding demographic information, very little is available about the US sample. Therefore, we have no means to compare for potentially influencing factors, like educational level and socio-economic status. Nevertheless we were able to compare the samples for mean age and gender distribution: Gray’s sample is, on average, younger (m = 26 years, t(213) = 26.21, *p* < .001) and contained more male participants (about 50%). In our data, there was a significant correlation of age with mind ascription in the UWS condition (r(219) = − 0.33, *p* < 0.000) where the younger participants ascribed more mind but no differences in mind ascription according to gender. This might suggest that, with our sample being older on average, and younger participants ascribing more mind, we might even have underestimated the differences in mind ascription between the US and the German sample. Additionally, in our sample, the effect of age was not mediated through religiosity or the fact, that younger participants might have easier access to information about UWS since we found no correlation between the self assed religiosity of participants and age (r(221) = .122, *p* > .05) or between the knowledge about UWS and age (r(218) = .129, *p* > .05).

## Conclusion

In sum, our data demonstrates that within the German sample, participants tend to ascribe mind to a UWS patient. In detail, German participant ascribe ‘emotions and feelings’ as well as a ‘personality’ to the UWS patient. Nevertheless, perception of UWS also varies greatly within the German sample. The presently assessed factors were able to account of 15% of the variance. However, the observed differences between the German and the US sample are consistent with important cross-cultural differences in the perception of UWS, the German participants ascribing more mind to UWS patients. Mind ascription reduces the risk of the patients to become objectified and therefore be denied moral rights [8]. Furthermore, if people ascribe mental abilities, patients are much more likely to be seen as persons, and by definition, a person (in contrast to a non-person) can be wronged [6].

This is important, because, in times of declining financial support for the healthcare systems, and an increasing legalization of euthanasia [30], it depends on the public whether ‘the right to die’ will be the only right conceded to UWS patients. However, given previous results on brain functions [19, 50–52] as well as unexpected late recovery [26, 57], there should also be other rights conferred to patients. Those rights could include, but are not limited to, the right of a correct diagnosis, the right for an empirically tested prognosis, the right for treatment and the right for inclusion into activities of daily living [29].

## Additional files

Additional file 1: **Table S1**. Cultural dimensions, key differences and ranking of the USA and Germany; Cultural dimensions according to Hofstede, the key differences of Germany and USA as well as the ranking of the two countries in comparison to 76 countries. (DOCX 12 kb)

Additional file 2: **Text S1**. Original and translated scenarios, Original David scenarios used in Gray et al. ‘More dead than dead’, 2011, supplemental material and the German online survey as implemented on ‘Unipark’. (DOCX 423 kb)

Additional file 3: **Text S2**. Translation of the German online survey; Translation of the German online survey. (DOCX 13 kb)

## Abbreviations

DOC: Disorders of consciousness
TBI: Traumatic brain injury
UWS: Unresponsive wakefulness syndrome
VS: Vegetative state
