# Supplementary material for: The living dead? Perception of persons in the unresponsive wakefulness syndrome in Germany compared to the USA
Source: BMC Psychol. 2018 Feb 21;6:5. doi: 10.1186/s40359-018-0217-4 (PMC5822482; doi:10.1186/s40359-018-0217-4)
Supplement: Supplementary file 1 — Table S1. Cultural dimensions, key differences and ranking of the USA and Germany; Cultural dimensions according to Hofstede, the key differences of Germany and USA as well as the ranking of the two countries in comparison to 76 countries. (DOCX 12 kb) [file 40359_2018_217_MOESM1_ESM.docx]

S 1 Table: Cultural Dimensions, key differences and ranking of the USA and Germany

| **Dimensions** | **Key differences** | | **Ranking**  of 76 countries |
| --- | --- | --- | --- |
| **Power Distance** | The extent to which the less powerful members of institutions and organizations within a country expect and accept that power is distributed unequally. | | USA: 59  Germany: 65  (both countries low power distance) |
| **Masculinity - Femininity** | A SOCIETY is called masculine when gender roles are clearly distinct | A SOCIETY is called feminin when gender roles overlap: both genders are supposed to be modest, tender and concerned with quality of life. | USA: 19  Germany: 11  (both countries rather masculine) |
| **Individual - Collective** | Ties between individuals are loose: everyone is expected to look after him- or herself. Children learn to think in terms of 'I'. | People from birth onward are integrated into strong, cohesive in-groups, which throughout people's lifetime continue to protect them in exchange for unquestioning loyalty. | USA: 1  (individualistic)  Germany: 19  (middle individualistic) |
| **Avoidance of Uncertainty** | The extent to which the members of a culture feel threatened by ambiguous or unknown situations, resulting (among others) in nervous stress and a need for predictability | | USA: 64  (low avoidance)  Germany: 43  (middle avoidance)  of 76 countries |
|  | Low: What is different is curious | High: What is different is dangerous |  |
| **Long-Term / Short-Term Orientation** | LTO: the fostering of virtues oriented toward future rewards - in particular, perseverance and thrift. | STO: the fostering of virtues related to the past and present - in particular, respect for tradition, preservation of 'face' and fulfilling social obligation and 'fun'. | USA: 69  (short-term orientation)  Germany: 6  (long term orientation)  of 93 countries |
| **Indulgence vs. Restraint** | Indulgence: a tendency to allow relatively free gratification of basic and natural human desire related to enjoying life and having fun. | Restraint: a conviction that such gratifications need to be curbed and regulated by strict norms | USA: 15 (indulgent)  Germany: 52  (restraint)  of 93 countries |
