# Supplementary material for: The living dead? Perception of persons in the unresponsive wakefulness syndrome in Germany compared to the USA
Source: BMC Psychol. 2018 Feb 21;6:5. doi: 10.1186/s40359-018-0217-4 (PMC5822482; doi:10.1186/s40359-018-0217-4)

S1 Text. Original and translated scenarios.

**Original David scenarios used in Gray et a., More dead than dead, supplemental material**

Appendix

Materials from Experiment 1

In Experiment 1, participants were presented with one of three vignettes in which the target either lived, died, or entered into a PVS. Participants were then asked to make ratings of mind.

Vignettes:

*Life* condition

David Tuchman grew up in a small city in Ohio. He went to college in Michigan and returned home to Ohio afterwards to work at his family’s local business. Shortly after he moved back home, he went out to dinner with some friends from high school at a local restaurant. On his way home from dinner, David’s car was struck head on by a truck that swerved across the median. David suffered from major injuries including temporary damage to his brain. He was in a coma for a short time but woke up. Now, David is fully recovered. His brain is fully functioning and he has all of the mental capacities of a normal person.

*Death* condition

David Tuchman grew up in a small city in Ohio. He went to college in Michigan and returned home to Ohio afterwards to work at his family’s local business. Shortly after he moved back home, he went out to dinner with some friends from high school at a local restaurant. On his way home from dinner, David’s car was struck head on by a truck that swerved across the median. When the ambulance arrived at the scene, there was nothing they could do to save him. David passed away two hours later at the hospital.

*PVS* condition

David Tuchman grew up in a small city in Ohio. He went to college in Michigan and returned home to Ohio afterwards to work at his family’s local business. Shortly after he moved back home, he went out to dinner with some friends from high school at a local restaurant. On his way home from dinner, David’s car was struck head on by a truck that swerved across the median. The ambulance arrived very quickly, but there was not much they could do for David. Although David did not die, he entered a Persistent Vegetative State. David’s entire brain was destroyed, except for the one part that keeps him breathing. So while his body is still technically “alive,” he will never wake up again.

German online Survey


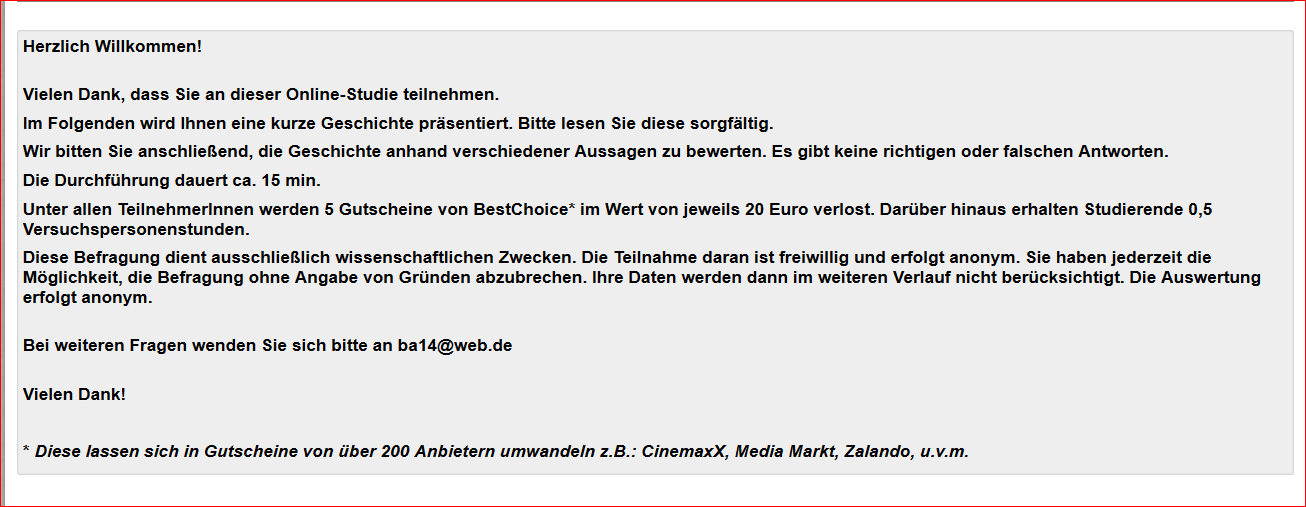


**Geschichte** (randomisierte Zuweisung)

Bitte lesen Sie die folgende Geschichte sorgfältig, da Sie anschließend einige Fragen dazu beantworten sollen. Sie können später nicht mehr auf die Geschichte zurückkommen.

**Leben-**Bedingung
David Tuchmann wuchs in einer kleinen Stadt in Bayern auf. Er ging in Hamburg zur Uni und kehrte danach nach Bayern zurück, um im Unternehmen seiner Familie zu arbeiten. Kurz nachdem er zurück nach Hause gezogen war, ging er mit ein paar Schulfreunden aus, um in einem Restaurant zu essen. Auf seinem Heimweg wurde Davids Auto frontal von einem LKW erfasst, der über die Mittellinie ausbrach. David erlitt schwere Verletzungen, unter anderem vorübergehende Hirnschäden. Er lag für kurze Zeit im Koma, erwachte jedoch wieder. Mittlerweile ist David vollkommen genesen, sein Gehirn funktioniert wieder vollständig und er weist alle mentalen Fähigkeiten einer normalen Person auf.

**PVS-**Bedingung
David Tuchmann wuchs in einer kleinen Stadt in Bayern auf. Er ging in Hamburg zur Uni und kehrte danach nach Bayern zurück, um im Unternehmen seiner Familie zu arbeiten. Kurz nachdem er zurück nach Hause gezogen war, ging er mit ein paar Schulfreunden aus, um in einem Restaurant zu essen. Auf seinem Heimweg wurde Davids Auto frontal von einem LKW erfasst, der über die Mittellinie ausbrach. Der Notarzt kam sehr schnell, aber er konnte nicht mehr viel für David tun. Zwar starb David nicht, aber er fiel ins Wachkoma. Davids Gehirn wurde vollständig zerstört, außer dem Teil, der ihn am Leben hält. Obwohl sein Körper technisch gesehen noch „lebt“, wird er nie wieder aufwachen.

**Tod-**Bedingung
David Tuchmann wuchs in einer kleinen Stadt in Bayern auf. Er ging in Hamburg zur Uni und kehrte danach nach Bayern zurück, um im Unternehmen seiner Familie zu arbeiten. Kurz nachdem er zurück nach Hause gezogen war, ging er mit ein paar Schulfreunden aus, um in einem Restaurant zu essen. Auf seinem Heimweg wurde Davids Auto frontal von einem LKW erfasst, der über die Mittellinie ausbrach. Als der Notarzt am Unfallort ankam, konnte er nichts mehr tun, um David zu retten. David starb zwei Stunden später im Krankenhaus.


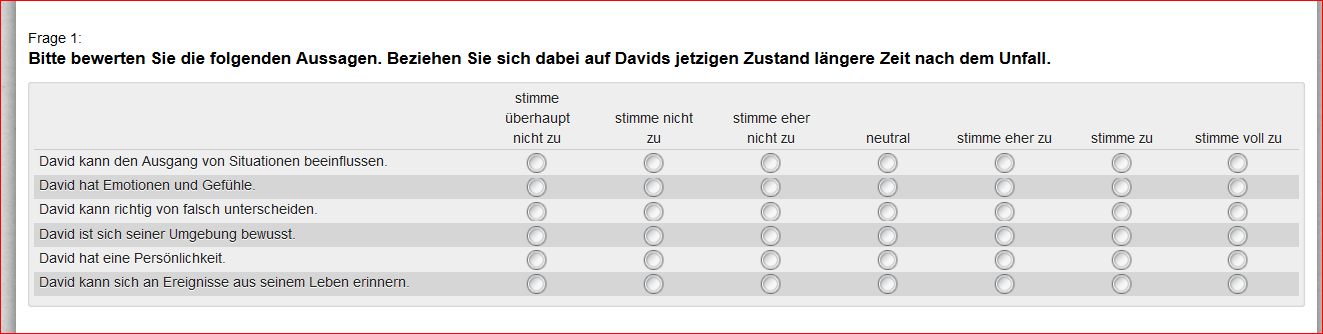


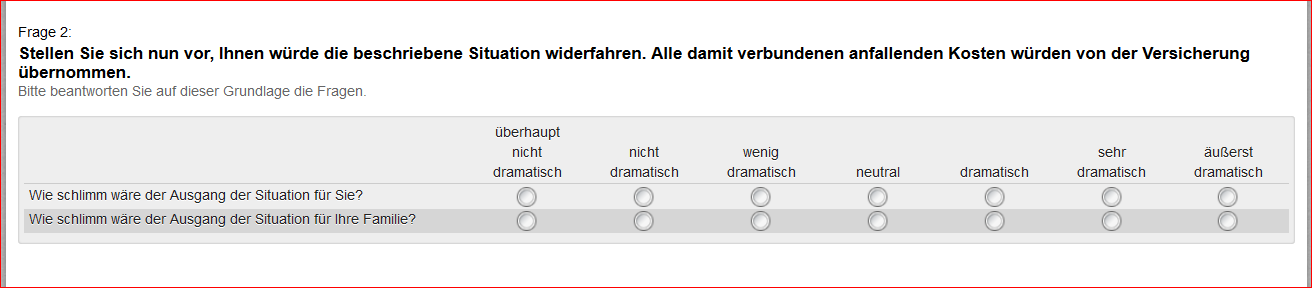

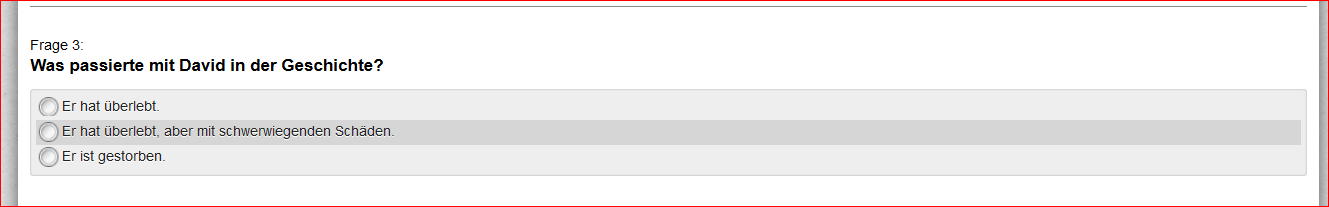

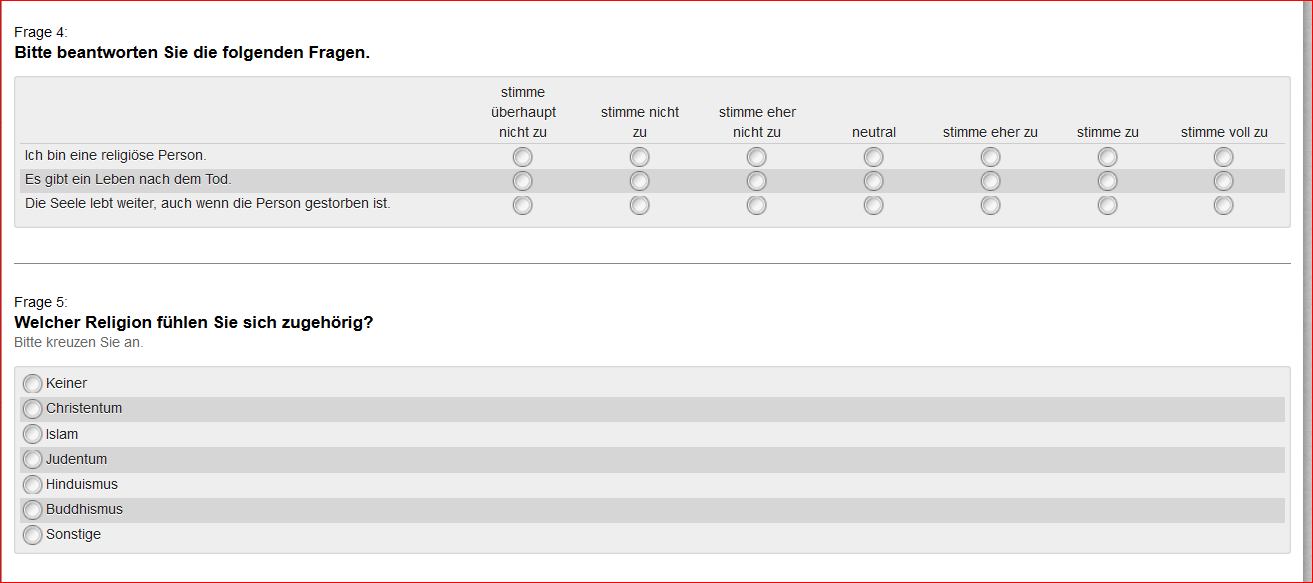

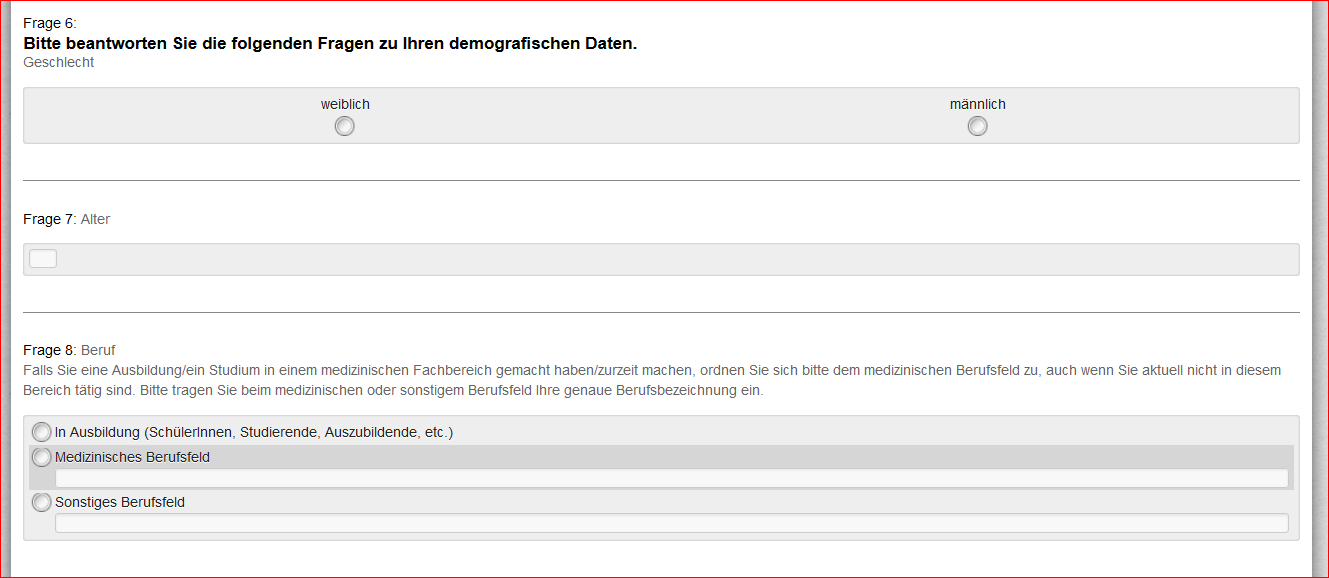

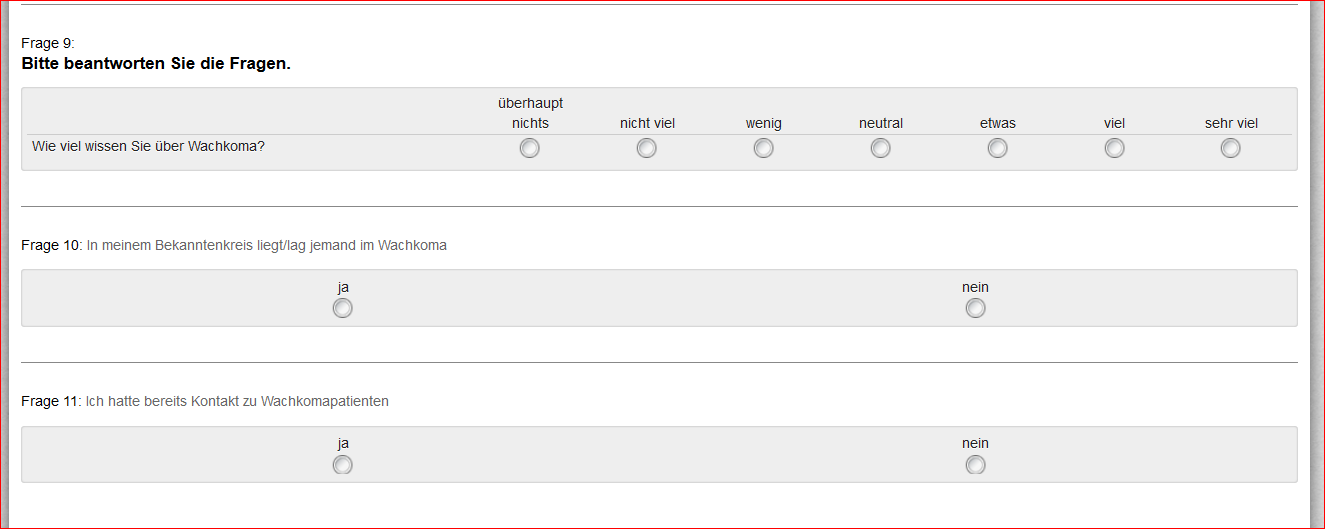

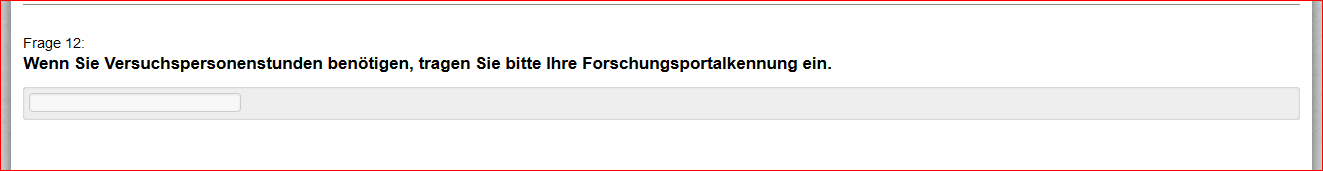

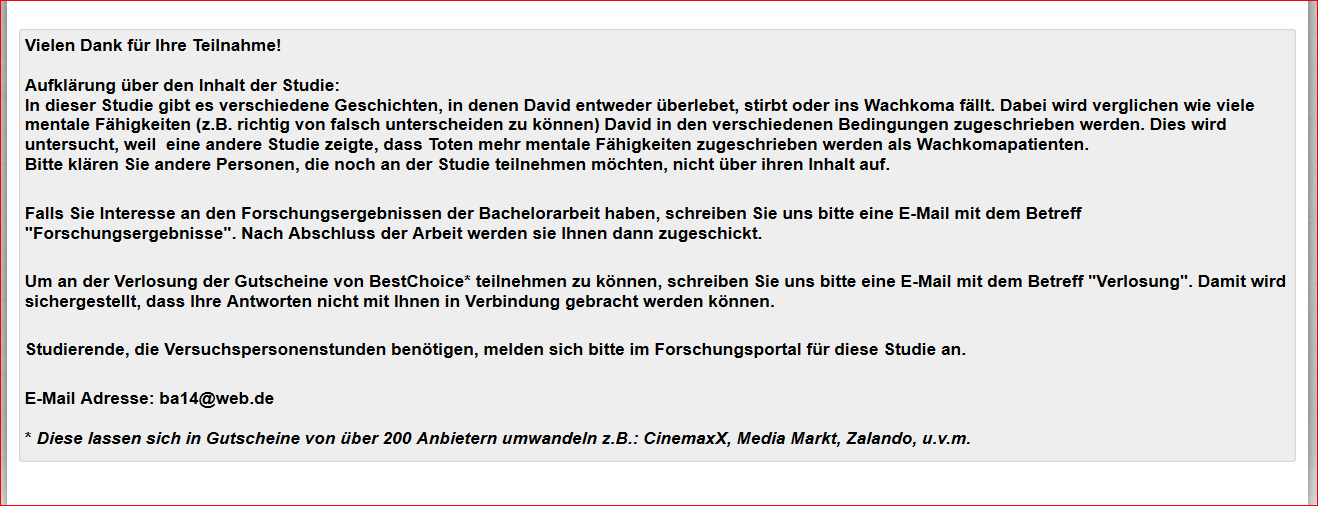

Supplement: Supplementary file 2 — Text S1. Original and translated scenarios, Original David scenarios used in Gray et al. ‘More dead than dead’, 2011, supplemental material and the German online survey as implemented on ‘Unipark’. (DOCX 423 kb) [file 40359_2018_217_MOESM2_ESM.docx]
