# Supplementary material for: The living dead? Perception of persons in the unresponsive wakefulness syndrome in Germany compared to the USA
Source: BMC Psychol. 2018 Feb 21;6:5. doi: 10.1186/s40359-018-0217-4 (PMC5822482; doi:10.1186/s40359-018-0217-4)
Supplement: Supplementary file 3 — Text S2. Translation of the German online survey; Translation of the German online survey. (DOCX 13 kb) [file 40359_2018_217_MOESM3_ESM.docx]

**S2 Text**

Translation of the German online survey

**Welcome!**

**Thank you for participating in this online survey.**

**In this experiment you will read a short story. Please read it carefully.**

**Later we will ask you to evaluate the story based on several statements. There are no right or wrong answers.**

**Participation will take about 15 minutes.**

**All participants will take part in a lottery. You can win one of 5 'BestChoice' vouchers worth 20 Euro each. If you are a student you will also be granted 0.5 participating hours.**

**This survey is solemnly used for research purpose. Participating is voluntary and anonym. You can stop or interrupt your participation at any point. Your data will then be excluded from analysis. Analysis is always anonym.**

**If you have questions of any kind you can contact ba14@web.de**

**Thank you very much!**

**Stories** (in randomized order)

Please read the following story carefully in order to answer some questions about it. You will not be able to return to this page later.

***Life* condition**

David Tuchman grew up in a small city in Bayern. He went to college in Hamburg and returned home to Bayern afterwards to work at his family’s local business. Shortly after he moved back home, he went out to dinner with some friends from high school at a local restaurant. On his way home from dinner, David’s car was struck head on by a truck that swerved across the median. David suffered from major injuries including temporary damage to his brain. He was in a coma for a short time but woke up. Now, David is fully recovered. His brain is fully functioning and he has all of the mental capacities of a normal person.

***Death* condition**

David Tuchman grew up in a small city in Bayern. He went to college in Hamburg and returned home to Bayern afterwards to work at his family’s local business. Shortly after he moved back home, he went out to dinner with some friends from high school at a local restaurant. On his way home from dinner, David’s car was struck head on by a truck that swerved across the median. When the ambulance arrived at the scene, there was nothing they could do to save him. David passed away two hours later at the hospital.

***Corps condition***

David Tuchman grew up in a small city in Bayern. He went to college in Hamburg and returned home to Bayern afterwards to work at his family’s local business. Shortly after he moved back home, he went out to dinner with some friends from high school at a local restaurant. On his way home from dinner, David’s car was struck head on by a truck that swerved across the median. David died on impact. After being embalmed at the morgue, he was buried in the local cemetery. David now lies in a coffin underground.

***PVS* condition**

David Tuchman grew up in a small city in Bayern. He went to college in Hamburg and returned home to Bayern afterwards to work at his family’s local business. Shortly after he moved back home, he went out to dinner with some friends from high school at a local restaurant. On his way home from dinner, David’s car was struck head on by a truck that swerved across the median. The ambulance arrived very quickly, but there was not much they could do for David. Although David did not die, he entered a Persistent Vegetative State. David’s entire brain was destroyed, except for the one part that keeps him breathing. So while his body is still technically “alive,” he will never wake up again.

**Question 1:**

Please evaluate the following statement: Please refer to David's state some time after the accident.

David can influence the outcome of situations

David has emotion and feelings

David knows right from wrong

David is aware of his environment

David possesses a personality.

David remembers life events

(Answer format: (1) strongly disagree - (2)disagree - (3)rather disagree - (4)neither agree nor disagree -(5) rather agree - (6)agree - (7)strongly agree)

**Question 2:**

Please imagine the described situation would happen to you. Insurance will pay in full for all costs.

How bad would this outcome be for you?

How bad would this outcome be for your family?

(Answer format: (1) not bad at all - (2)not so bad - (3)not bad - (4)neutral -(5) bad- (6) very bad (7)extremely bad)

**Question 3:**

What happened to David in the story?

(Answer format: He survived; He survived but with severe brain damage; He died)

**Question 4:**

Please answer the following questions:

I am a religious person

There is life after death

The soul lives on even after the person has died.

(Answer format: (1) strongly disagree - (2)disagree - (3)rather disagree - (4)neither agree nor disagree -(5) rather agree - (6)agree - (7)strongly agree)

**Question 5:**

Which religion do you belong to?

(Answer format: None - Christians - Buddhist - Muslim - Hindus - Jewish - Other)

**Questions 6 and 7:**

Your gender? female / male

How old are you?

**Question 8:**

Your area of work? (Student / Medical background (please specify)/ Other work area (please specify)

**Question 9:**

How much do you think you know about waking coma? (nothing at all - a little bit - not much - neutral - some - much - very much)

**Question 10 and 11:**

I have a waking coma patient within my circle of acquaintances (Yes / No)

I have/had contact to a patient in a waking coma (Yes / No)

**Question 12:**

If you need participation hours please enter your code here:

**Thank you very much for your participation!**

**Clarification about the content of this study:**

**In this study you read one of three stories about David who either survived, died or entered a waking coma. We were interested in the amount of mental abilities ascribed to David in each scenario. With that, we try to replicate a study which found that participants ascribed more mind to the deceased David than to David in a waking coma. Please do not inform other participants about the purpose of this study who have not finished the survey yet!**

**If you are interested in our results, please send an Email with the reference 'Results'. We will answer your email after the study is closed.**

**To enter the lottery, please send an email with the reference 'Lottery'. This way we can ensure that your data and your email-address will be stored separately.**

**Send your email to: ba14@web.de**
